# Ionic liquid-based dispersive liquid-liquid microextraction combined with functionalized magnetic nanoparticle solid-phase extraction for determination of industrial dyes in water

**DOI:** 10.1038/s41598-017-14098-1

**Published:** 2017-10-23

**Authors:** Ning Liang, Xiaohong Hou, Peiting Huang, Chao Jiang, Lijuan Chen, Longshan Zhao

**Affiliations:** 1 School of Pharmaceutical Engineering, Shenyang Pharmaceutical University, Shenyang Liaoning Province, 110016 P. R. China; 2 School of Pharmacy, Shenyang Pharmaceutical University, Shenyang Liaoning Province, 110016 P. R. China

## Abstract

N-butyl pyridinium bis((trifluoromethyl)sulfonyl)imide ([Hpy]NTf_2_) functionalized core/shell magnetic nanoparticles (MNPs, Fe_3_O_4_@SiO_2_@[Hpy]NTf_2_)) were prepared and applied as an adsorbent for magnetic solid phase extraction (MSPE) of three commonly used industrial dyes including malachite green, crystal violet and methylene blue. Extraction solution was mixed with 100 mg extraction material of Fe_3_O_4_@SiO_2_@[Hpy]NTf_2_, and 1 mL of acetonitrile was used to elute target analytes for further extraction and purification. [Hpy]NTf_2_ was used as extraction solution, and 500 μL methanol was selected as dispersive solvent in ionic liquid (IL) dispersive liquid–liquid microextraction (DLLME) method. After sonication for 5 min and centrifugation at 447 g for 10 min, 20 μL of sedimented phase was injected into HPLC-UV system. The limit of detection (LOD) and limit of quantification (LOQ) of current method were 0.03 and 0.16 μg·L^−1^, respectively, which indicated the sensitivity was comparable or even superior to other reported methods. The relative recoveries of the target analytes ranged from 86.1% to 100.3% with relative standard deviations between 0.3% and 4.5%. The developed method has been successfully applied to determine the level of three industrial dyes in different water samples.

## Introduction

Malachite green (MG), crystal violet (CV) and methylene blue (MB) are three commonly used compounds with a broad range of applications in aquatic product industry as antibiotics and antiparasities, also in industry as dyes used in the textile process^1–3^. Those dyes have been known as genotoxic and carcinogenic properties, which can induce hepatic and renal tumors in mice and reproductive abnormalities in fish, along with the risk of human bladder cancer^4,5^. Widely used MG, CV and MB in diverse aquatic products and textile leads to its presence greater or lesser concentrations in drinking water. Considering of the serious adverse effects on human health, it is important to develop a simple, rapid, and sensitive method for simultaneous trace determination of three dyes in water. However, such dyes are usually present in low concentration in real samples, preconcentration and clean-up steps are necessary for reliable determination of these compounds prior to the analysis.

The most widely used pretreatment methods for isolation and enrichment of MG, CV or MB in aquatic products and water include solid-phase extraction (SPE)^6,7^, molecularly imprinted solid phase extraction (MISPE)^8,9^, magnetic solid-phase extraction (MSPE)^10,11^, dispersive liquid-liquid microextraction (DLLME)^12,13^, etc. MSPE is a procedure for preconcentration of target analytes from large volumes based on the use of magnetizable sorbents, which can be easily separated from sample solutions by an external magnetic field without additional centrifugation or filtration. Furthermore, the exposed surface between analytes and sorbents is so large that the sorbents can be dispersed in sample solution easily, resulting rapid mass transfer and extraction equilibrium. Ionic liquid (IL) has been used as green solvents instead of traditional organic solvents because of its special chemical and physical properties, such as high polarity and thermal stability, low volatility and wonderful miscibility. Recently there are several novel sorbents using ionic liquids coated magnetic nanoparticles have been applied for trace enrichment of drug residues in water samples^14,15^. DLLME is a microextraction technique applying appropriate extraction solvent and dispersant for the microextraction of analytes in aqueous sample with merits of rapidity, low cost, high recovery and simplicity of operation. However, there are some limitations when using DLLME such as highly toxic extractive and dispersive solvents. To overcome these difficulties, the usage of ILs has been applied as green solvent to replace the conventional organic solvents to extract pollutants which has shown great interests. There are several reports using ionic liquid based DLLME for trace enrichment of organic chemicals in foods, biological, and water samples^16–18^. In our previous investigations, we developed the method which combined SPE with DLLME (SPE-DLLME) as an efficient hyphenated technique so as to successfully improve the selectivity and sensitivity in pretreatment process^13,19,20^.

In the present study, a novel kind of MNPs termed as N-butyl pyridinium bis((trifluoromethyl)sulfonyl)imide ([Hpy]NTf_2_) functionalized magnetic silica nanoparticles (Fe_3_O_4_@SiO_2_@[Hpy]NTf_2_) has been developed to extract MG, CV and MB from water samples. [Hpy]NTf_2_ has been applied as extraction solution for further purification in IL-DLLME method. To the best of our knowledge, this is the first report about preparation of ionic liquid based magnetic nanoparticles (MNPs), and their applications of SPE sorbents on simultaneous adsorption of MG, CV and MB in water.

## Experimental

### Reagents and materials

MG (purity > 98%), CV (purity > 98%) and MB (purity > 98%) were purchased from the National Institute for Food and Drug Control (Beijing, China). N-butyl pyridinium bis((trifluoromethyl)sulfonyl)imide ([Hpy]NTf_2,_ purity > 99%) was obtained from Shanghai Chengjie Chemical Co., Ltd (Shanghai, China). Iron oxide (100 nm, purity > 98%) was purchased from Aladdin Industrial Co., Ltd (Shanghai, China). Tetrathoxysilane (TEOs, purity > 99%) was purchased from Sinopharm Chemical Reagent Co., Ltd (Shanghai, China). Acetonitrile and formic acid (chromatographic grade) were obtained from Yuwang Industrial Co., Ltd (Shandong, China). All other chemicals and solvents in this experiment including dichloromethane (CH_2_Cl_2_), dichloroethane (C_2_H_4_Cl_2_), trichloromethane (CHCl_3_), carbon tetrachloride (CCl_4_), chlorobenzene (C_6_H_5_Cl) were analytical grade. Deionized water was used throughout the experiments. The cartridges used for solid phase purification were Cleanert Alumina N (1000 mg, 6 mL) which was purchased from Agela Technologies (Tianjin, China).

The drinking water was obtained from bottled water in Shenyang and samples of running water were taken from the tap in the laboratory. River water (RW) samples were collected from the South Canal of Shenyang. Influent (IWW) and effluent (EWW) wastewaters were collected from the urban wastewater treatment plant of Shenyang. All samples were collected in December 2015 and filtered by 0.45 μm nylon membrane and stored in amber glass bottles at −20 °C before analysis.

### Instrumentations and analytical conditions

An LC-20AT liquid chromatography equipped with a UV detector (Shimadzu Co., Ltd., Kyoto, Japan) was used for the chromatographic analysis and the detection wavelength was 615 nm. Separations were performed on a Kromasil C_18_ column (250 mm × 4.6 mm, 5 μm) and the column temperature was maintained at 30 °C. A good chromatographic separation was implemented with a mobile phase consisting of acetonitrile and 0.1% formic acid in water solution (60:40, *v/v*) at a flow rate of 1 mL·min^−1^. An X’Pert PRO X-ray diffractometer (PANalytical Co., Netherlands), an HCT-1 TA instruments (Beijing Hengjiu Scientific Instrument Factory, Beijing, China), an SSX-550 SEM instruments (Shimadzu Co., Ltd., Kyoto, Japan) and a Waters 2414 Infrared spectrometer (Waters Corp., Milford, MA, USA) were conducted for characterization of the materials. A KQ5200DE ultrasonic cleaner bath (Kunshan ultrasonic instrument co., Ltd., Kunshan, China) and JJ-1 electric mixer (Jiangsu Ronghua instrument manufacturing co., Ltd., Jiangsu, China) were used for synthesis of the materials. A pH meter (PHS-3CF, Shanghai, China) was employed for pH adjustment. A vortex mixer (XW-80A, Jiangsu, China) was used for thoroughly mixing the solution.

### Preparation of Fe_3_O_4_@SiO_2_@[Hpy]NTf_2_ MNPs

#### Preparation of Fe_3_O_4_@SiO_2_ MNPs

1.0 g Fe_3_O_4_ was added to a round-bottom flask which contained 50 mL ethanol and 15 mL pure water. The mixture was sonicated for 15 min to ensure homogeneous mixture. After adjusting the pH to 9 using ammonia, 2 mL of TEOs was added dropwise to the flask. The mixture was mechanically stirred under nitrogen protection for 24 h to perform the silica coating. The produced Fe_3_O_4_@SiO_2_ was separated and washed with deionized water, to which was added 1 mL HCl solution (1 mol·L^−1^) and mechanically stirred for 12 h at room temperature. The MNPs were collected by a magnet, washed with water, and vacuum-dried at 60 °C for 12 h.

#### Synthesis of Fe_3_O_4_@SiO_2_@[Hpy]NTf_2_

1.0 g of [Hpy]NTf_2_ was dissolved in 10 mL acetone, followed by addition of 1.0 g Fe_3_O_4_@SiO_2_. The mixture was stirred for 30 min, and the sample was dried under stirring and a stream of nitrogen purge. The resultant was washed with CH_2_Cl_2_, and dried at 60 °C.

### Extraction procedure

One hundred milligram of Fe_3_O_4_@SiO_2_@[Hpy]NTf_2_ was added to 100 mL of water sample (pH was adjusted to 4.0 by using HCl) in a conical flask. The mixture was shaken for 20 min and an external magnet was applied to isolate the material from the water. 1 mL of acetonitrile was used to elute target analytes from the material by shaking for 20 min. The elute solvent was transferred to a 10-mL glass conical tube after being separated by a magnet. The elution was evaporated to dry on a rotary vacuum evaporator at 35 °C, and 5 mL of pure water (adjusted pH to 4.0 using HCl) was added to the test tube to reconstitute the residue. A mixture of 70 μL of [Hpy]NTf_2_ and 500 μL of methanol was rapidly injected into the aqueous solution to form a cloudy solution, which was sonicated for 5 min to increase the extraction efficiency. The mixture was centrifuged at 447 g for 10 min and the dispersed fine particles of the extraction phase were deposited at the bottom of the test tube. The supernatant was removed using a microsyringe. The remaining sedimented phase was diluted by mobile phase solution (acetonitrile/water, *v/v*, 60/40), 20 μL of which was injected into HPLC system for analysis.

## Results and Discussion

### Characterization of Fe_3_O_4_@SiO_2_@ [Hpy]NTf_2_ MNPs

The prepared Fe_3_O_4_@SiO_2_@[Hpy]NTf_2_ MNPs were characterized with FTIR spectroscopy, thermogravimetric analysis (TGA), X-ray diffraction (XRD) and scanning electron microscope (SEM).

**Figure 1:**
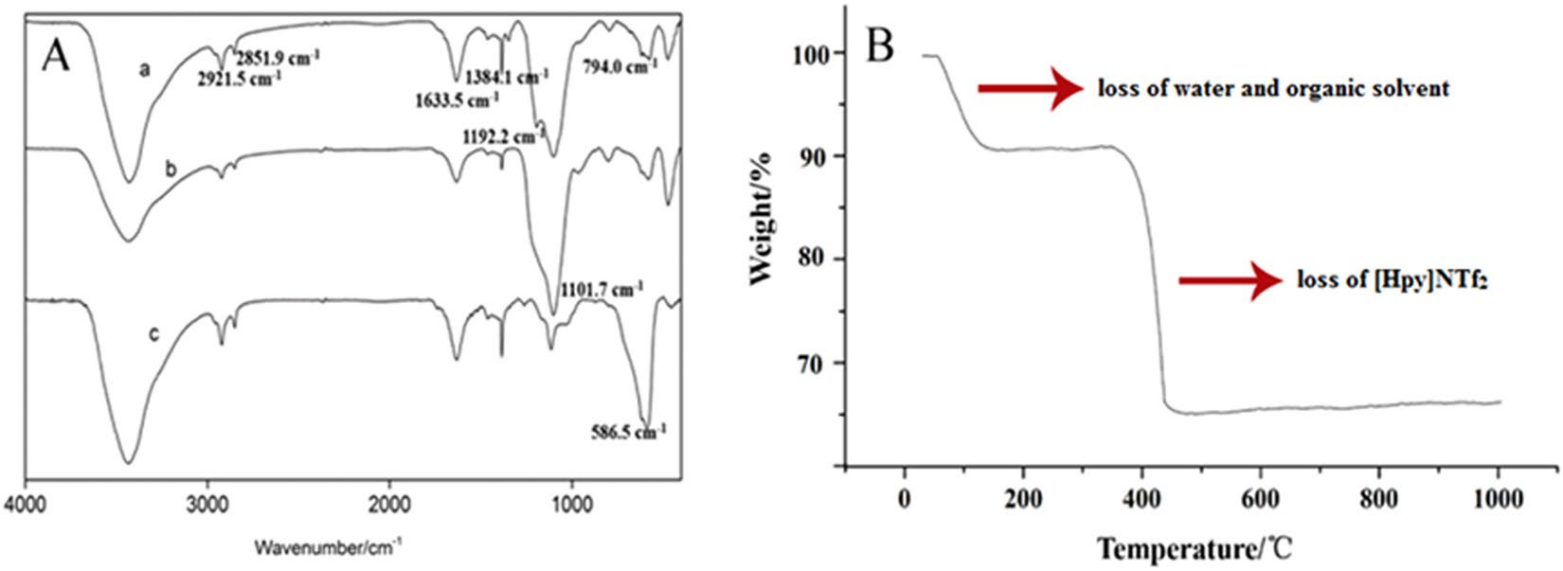
FTIR spectra of (**A**) Fe_3_O_4_@SiO_2_@[Hpy]NTf_2_ (a), Fe_3_O_4_@SiO_2_ (b), Fe_3_O_4_ (c) (**B**) TGA of Fe_3_O_4_@SiO_2_@[Hpy]NTf_2_.

As shown in Fig. 1A, the appearance of Fe_3_O_4_@SiO_2_@[Hpy]NTf_2_ (a) characteristic peaks at 2921.5 and 2851.9 cm^−1^ were attributed to the C-H asymmetrical stretching vibration, 1633.5 cm^−1^ for the C=N stretching vibration, 1384.1 cm^−1^ for -CH_3_ bending asymmetrical vibration, 1192.2 cm^−1^ for C-H flexural vibration, and 794.0 cm^−1^ for C=C stretching vibration, respectively. Comparing curves between Fe_3_O_4_@SiO_2_ (b) and Fe_3_O_4_ (c), prominent peaks at 586.5 cm^−1^ (Fe-O-Fe) disappeared and there was a peak shown at 1101.7 cm^−1^ which was related to Si-O-H and Si-O-Si, all of these results demonstrated the successful surface modification of the MNPs.

In this study, TGA was conducted in a nitrogen atmosphere, and the temperature was increased from 25 to 1000 °C at a rate of 5 °C·min^−1^. As can be seen in Fig. 1B, an additional weight loss of 8% after heating to 150 °C, corresponded to water and organic solvent content. Besides, an obvious weight loss of 25% was observed from 400 to 600 °C due to the decomposition of the ILs, which was consistent with the decomposition temperature of Bis(trifluoromethane sulfonimide) ILs^21^. This observation indicated that [Hpy]NTf_2_ was located on the surface of Fe_3_O_4_@SiO_2_. There was no significant weight loss when the temperature increasing up to 500 °C, which indicated the yield of final products was about 70%.

**Figure 2:**
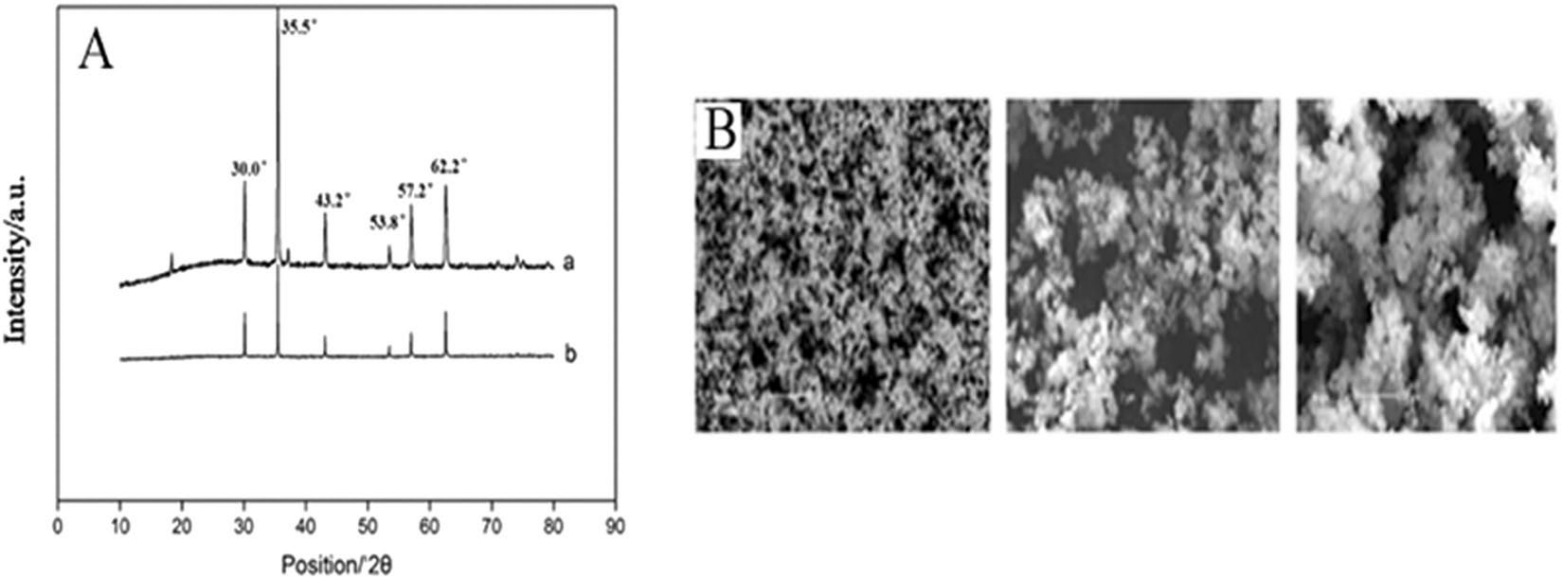
(**A**) XRD of Fe_3_O_4_@SiO_2_ (a) and Fe_3_O_4_@SiO_2_@[Hpy]NTf_2_ (b); (**B**) SEM of Fe_3_O_4_ (a), Fe_3_O_4_@SiO_2_ (b), Fe_3_O_4_@SiO_2_@[Hpy]NTf_2_ (c).

Figure 2A showed the XRD of Fe_3_O_4_@SiO_2_ (a) and Fe_3_O_4_@SiO_2_@[Hpy]NTf_2_ (b). There were 6 characteristic diffraction signals at 2θ = 30.0°, 35.5°, 43.2°, 53.8°, 57.2°, 62.2° which were related to Fe_3_O_4_. Furthermore, there was a smooth diffraction peak at 2θ = 20.0° corresponding to the amorphous structure of the SiO_2_ nanomaterials. Which illustrated the appearance of Fe_3_O_4_@SiO_2_. Comparing with curve a, the intensity of all signals was decreased in curve b which was caused of ionic liquid coated on the surface of Fe_3_O_4_@SiO_2_. The spectrum of XRD indicated that synthesized Fe_3_O_4_@SiO_2_ nanoparticles contained both Fe_3_O_4_ and SiO_2_ crystal structure, and [Hpy]NTf_2_ was successfully coated on Fe_3_O_4_@SiO_2_.

Scanning electron microscope (SEM) produces microscopic images of sample surface material. As shown in Fig. 2B, these are SEM pictures of Fe_3_O_4_, Fe_3_O_4_@SiO_2_, and Fe_3_O_4_@SiO_2_@[Hpy]NTf_2_. SEM spectrum of Fe_3_O_4_ shows small particle size, spherical shape, no accumulation, dispersed particles, and 100 nm-pore size, which are consistent with the parameters in label of commercial product. The particle size of Fe_3_O_4_@SiO_2_ is about 200 nm, which is larger than that of Fe_3_O_4_. Most of the particles are spherical, but the size is large which may due to poor grinding and dispersion forming during synthesis process. The particle size of Fe_3_O_4_@SiO_2_@[Hpy]NTf_2_ is between 200~800 nm, but aggregated which affects the particle size. It is due to high viscosity of ionic liquid, which can be further demonstrated that the synthesized material is Fe_3_O_4_@SiO_2_@[Hpy]NTf_2_.

### Optimization of chemical synthesis

#### Chemical synthesis of Fe_3_O_4_@SiO_2_

When large amount of TEOs was added to the reaction system, most of the synthesized SiO_2_ would disperse into the solution. This was because of limited binding sites on the surface of Fe_3_O_4_, whereas extra TEOs would lead to more SiO_2_ into the free state. The amount of TEOs was also optimized and results indicated that 2 mL of TEOs was the most suitable volume which was applied in the further investigation. Besides, pH showed great influence on the productivity. When the pH was below 9, the final productivity was extremely low, however when pH was adjusted to 9–10, productivity was improved from 40% to 70%. This indicated the reaction should be proceeded under basic conditions, because hydroxyl group (-OH) group could provide binding sites for SiO_2_. Insufficient basicity could interfere the number of binding sites, whereas the basicity showed no influence on the binding sites when it was saturated.

#### Chemical synthesis of Fe_3_O_4_@SiO_2_@[Hpy]NTf_2_

Acetone was used as the reaction solvent, while volume could directly affect the synthesis efficiency, and therefore, 5, 10, 15, 30 mL of acetone was investigated under the conditions of both Fe_3_O_4_@SiO_2_ and [Hpy]NTf_2_ applying 1.0 g. The results indicated that the highest synthesis efficiency of 64.96% was achieved with the volume of 10 mL acetone. Therefore, 10 mL acetone was used in the further investigation.

The ratios of Fe_3_O_4_@SiO_2_ and [Hpy]NTf_2_ were selected as 2:10, 3:10, 5:10, 10:10, 15:10, 20:10 (m:m) so as to investigate the best ratio for synthesis. The results indicated that the productivity and availability of [Hpy]NTf_2_ were 64.96% and 30.14% when using the ratio of 10:10, so the final amount of both Fe_3_O_4_@SiO_2_ and [Hpy]NTf_2_ were 1.0 g.

When the temperature was below 25 °C, ILs could not be efficiently attached on the surface of Fe_3_O_4_@SiO_2_. However, productivity will not increase above 30 °C, with the possible reasons of molecular thermal movement; therefore, 25 °C was selected as the reaction temperature.

### Optimization of MSPE

The effects of several parameters on extraction efficiency by MSPE were studied, including pH, sorbent amount, extraction time, type of desorptive solvent and volume. The influence of all these parameters was evaluated in terms of peak areas.

#### Effect of pH

**Figure 3:**
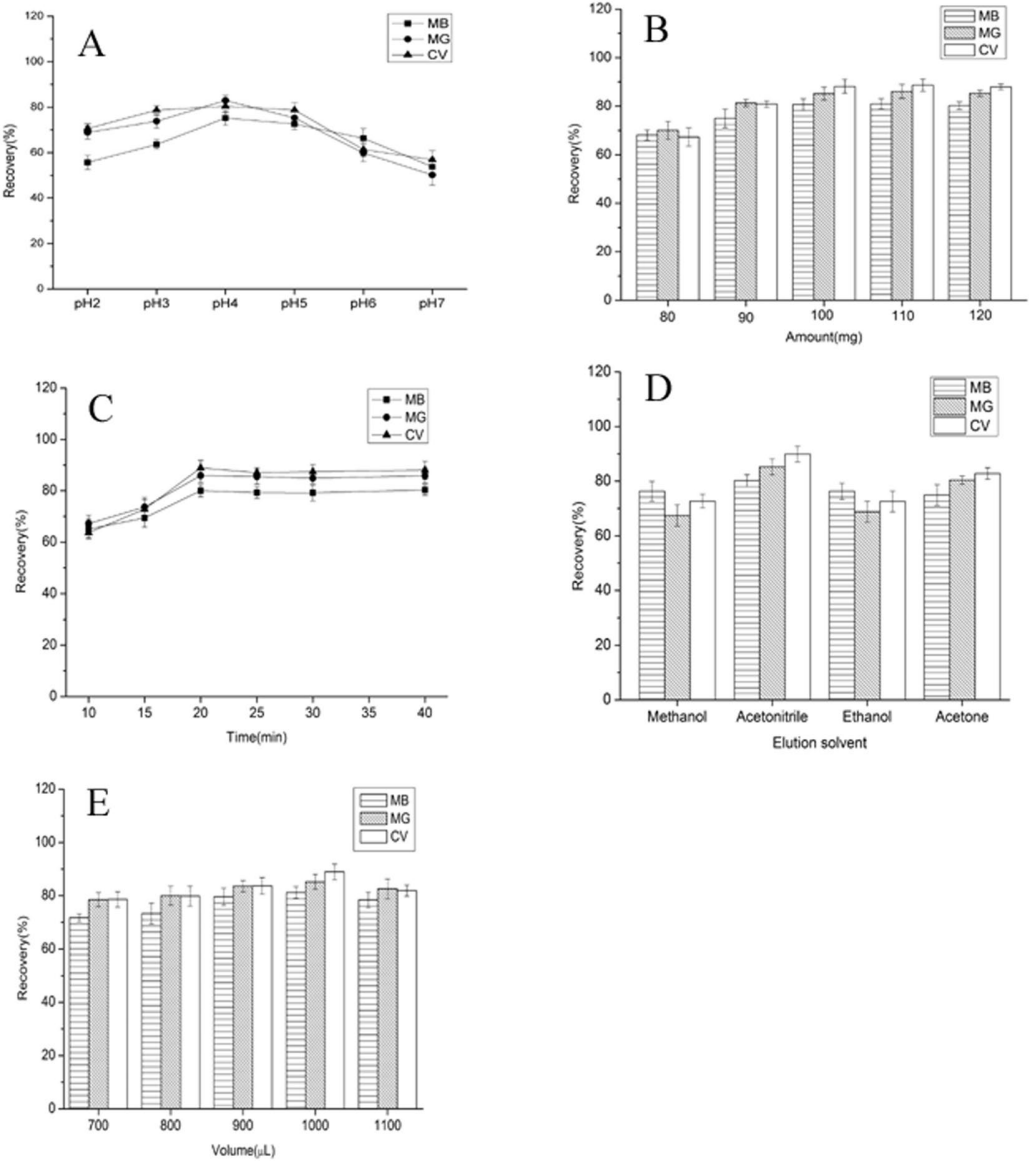
(**A**) Effect of pH of MSPE on the recoveries of targets; (**B**) Effect of amount of material on the recoveries of targets; (**C**) Effect of time of MSPE on the recoveries of targets; (**D**) Effect of elution solvent on the recoveries of targets; (**E**) Effect of volume of elution solvent on the recoveries of targets.

The sample pH is a crucial factor in the extraction process which could affect the existing state of the targets and extraction efficiency. Considering the pKa values of 6.9, 0.8 and 4.5 for MG, CV and MB, respectively, the water samples should be acidic, so as to make the targets to be presented in molecular form in water samples. In this work, the effect of pH was investigated over the pH range of 2.0–7.0. As shown in Fig. 3A, the recoveries of three targets increased and reached a maximum value at a pH of 4.0, and subsequently decreased, which indicated that three targets were successfully extracted to the IL sediment phase. Therefore, pH 4.0 was selected for the subsequent assays.

#### Effect of amount

The effect of amount of Fe_3_O_4_@SiO_2_@[Hpy]NTf_2_ MNPs was investigated with the amount varying from 70 to 120 mg. Figure 3B demonstrated that the peak areas of almost all the 3 dyes increased continuously with increasing the sorbent amount from 80 to 100 mg. Further increasing the sorbent amount showed no obvious increasing in the peak area of the targets. These results indicated that 100 mg of sorbent was sufficient to extract dyes in water, and therefore, 100 mg of sorbent was used for the following experiments.

#### Effect of extraction time

To evaluate the effect of extraction time on the recoveries of targets, different extraction time ranging from 5 to 30 min were tested (Fig. 3C). The recoveries gradually increased up to 20 min, and slightly increased when extraction time was increased to 30 min. Thus, 20 min was selected as the optimal extraction time.

#### Effect of the type of elution and its volume

An appropriate type of elution solvent is important for perfect extraction efficiency by Fe_3_O_4_@SiO_2_@[Hpy]NTf_2_ MNPs. Four types of solvents were selected as desorptive solvent, including methanol, ethanol, acetonitrile and acetone. As shown in Fig. 3D, acetonitrile yields higher recovery than that of methanol, ethanol, and acetone. The effect of sample volume on extraction efficiency of targeted dyes was investigated with the volume changing from 700 to 1100 μL. As shown in Fig. 3E, the recoveries of all these three dyes declined with increasing the sample volume from 1000 to 1100 μL. This is because when increasing the sample volume, especially for 700–1000 μL, the magnetic sorbents would be dispersed more widely and sorbents lost as long as volume increased. Then, 1000 μL was selected as sample volume.

Based on the above discussions, the optimal conditions for enrichment of targets were determined as follows: Fe_3_O_4_@SiO_2_@[Hpy]NTf_2_ MNPs, 100 mg; sample pH, 4.0; extraction time, 20 min; desorption solvent and volume, 1000 μL acetonitrile.

### Optimization of DLLME

#### Effect of extraction solvent volume

**Figure 4:**
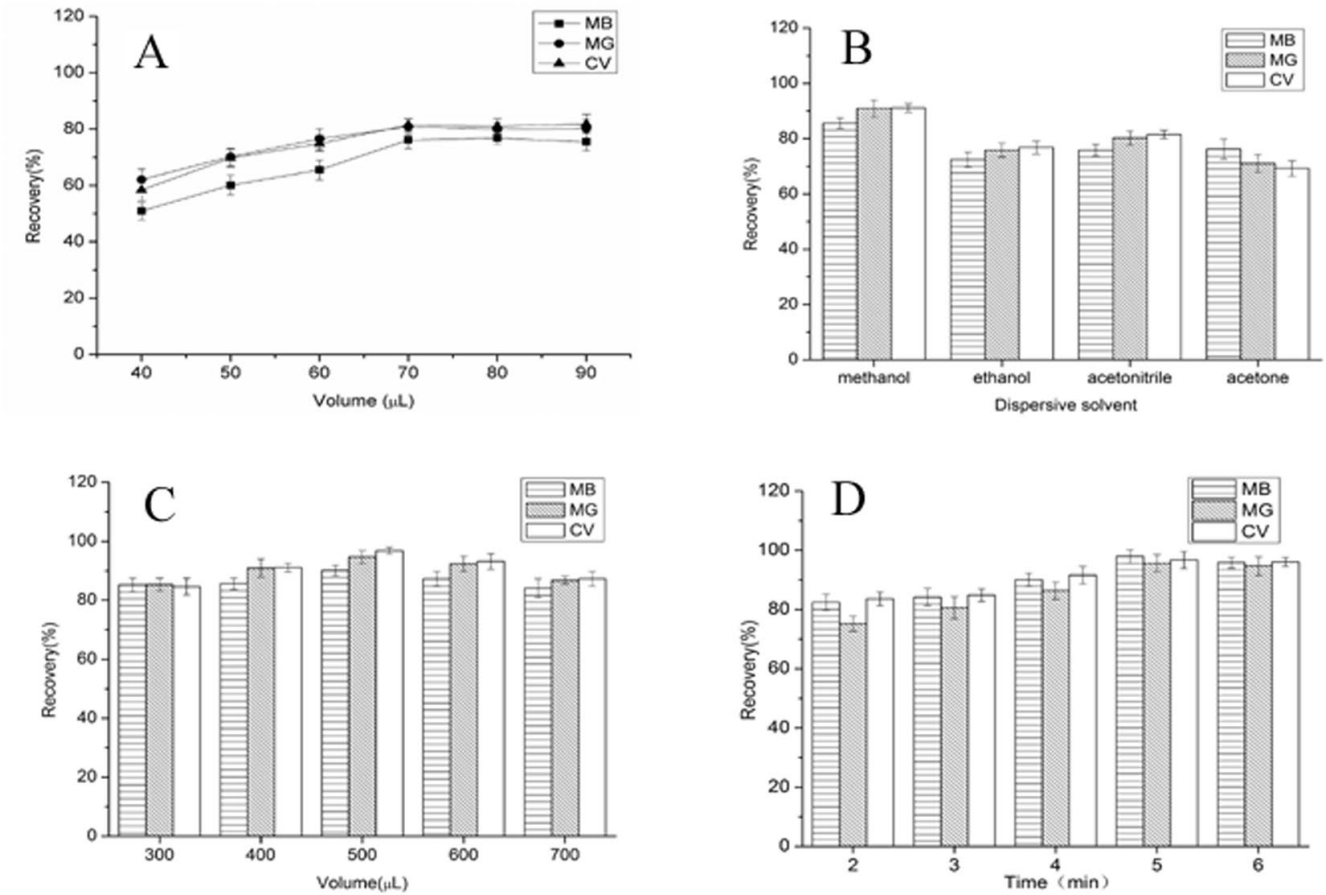
(**A**) Effect of extraction solvent volume of DLLME on the recoveries of targets; (**B**) Effect of dispersive solvent of DLLME on the recoveries of targets; (**C**) Effect of dispersive solvent volume of DLLME on the recoveries of targets; (**D**) Effect of ultrasound time of DLLME on the recoveries of target.

In order to evaluate the influence of extraction solvent volume on extraction efficiencies of targets, different volumes (40, 50, 60, 70, 80, 90 μL) of [Hpy]NTf_2_ were tested using the same MSPE-DLLME procedure. As shown in Fig. 4(A), the recoveries of targets increased with increasing the volume of [Hpy]NTf_2_ from 40 to 70 μL, but above 70 μL, the recoveries remained a constant level. Considering the enrichment factor decreased when the IL solvent volume was increased. Thus, 70 μL of [Hpy]NTf_2_ was selected in the following studies, since the higher recoveries were obtained and the EFs were acceptable.

#### Effect of dispersive solvent and its volume

The dispersive solvent should efficiently disperse the extraction solvent into the aqueous sample to increase the contact area. The main point to be considered in selecting dispersive solvent is its miscibility in both organic and aqueous phases. Methanol, ethanol, acetone and acetonitrile were widely used as dispersive solvents. Based on Fig. 4(B) we concluded that methanol gave the highest recoveries among the solvents. Thus, methanol was chosen as the disperser solvent.

In the meanwhile, the volume of disperser solvent affects the dispersion degree of the extraction solvent in aqueous phase, consequently affecting the extraction efficiency. Therefore, the effect of dispersive solvent volume on the extraction recovery was also investigated in the range of 0.4–1.2 mL. The obtained results (Fig. 4C) showed that the extraction recovery increased with the increasing volume of methanol up to 0.5 mL and slightly decreased as the volume increasing. Thus, 0.5 mL of acetonitrile was selected as the optimum volume.

#### Effect of ultrasound time

The effect of ultrasonic time on the extraction efficiency was examined within the range from 2 to 6 min. As shown in Fig. 4(D), ultrasonic time of 5 min was the optimal condition and longer ultrasonic time could not obtain higher extraction efficiency. When the mixture of ionic liquid extraction solvent and dispersive solvent were injected to the aqueous sample, the fine droplets of extraction solvent provided an infinitely large contact surface area between aqueous phase and extraction solvent to speed up the mass-transfer process. It would take a short period of time for the analytes to transfer from aqueous phase to extraction phase and to reach an equilibrium state. However, the volatilization of organic solvent and consequent thermal effect of analytes would lead to the loss of analytes as the reaction time went longer. Hence, 5 min was chosen as the optimum ultrasonic time.

#### Effect of ionic strength

The effect of ionic strength of the sample solution was evaluated by adding NaCl (0–10%, w/v) under the previous optimum conditions. Generally, addition of moderate salt could decrease the solubility of targets in the aqueous sample, thus increasing the extraction efficiency. However, in the current investigation, we concluded that ionic strength did not have significant effect on the extraction of target dyes. Therefore, the MSPE-IL-DLLME procedure was performed without salt addition to the sample solutions.

#### Analytical performance of the method

Under the optimized conditions, the method was applied to determination of dyes in water samples. The analytical results can be found in Table 1. The calibration curves were established by plotting peak area against concentration. The regression coefficients were determined to be 0.998. The limit of detection (LOD) and limit of quantification (LOQ) were 0.03–0.05 μg·L^−1^ and 0.11–0.16 μg·L^−1^, respectively. The precisions of the method ranged from 1.2% to 2.6% (n = 6) and accuracies varied between 0.3% and 4.5%, indicating that the method showed high sensitivity and reproducibility.

**Table 1:** The performance characteristics of MSPE-DLLME.

Analytes	Linear range (μg∙L^−1^)	*r*	LOD (μg∙L^−1^)	LOQ (μg∙L^−1^)	Precision RSD% (n = 6)	Repeatability RSD % (n = 6)	Enrichment Factor
MB	0.20–20	0.9993	0.05	0.16	1.2	2.4	502
MG	0.20–20	0.9986	0.03	0.11	2.6	3.8	484
CV	0.20–20	0.9991	0.04	0.13	1.9	3.4	499

#### Analysis of real water samples

To further evaluate the method applicability, South Canal river water, tap water, drinking water and waste water samples were analyzed using established method. Accuracy of a method was evaluated as the percentage of deviation from the known added amount of analyte in the sample. It is generally investigated by recovery experiments. Analyte recovery was calculated according to the equation as follows:

$${\rm{Rec}}=\frac{{\rm{C}}-{\rm{B}}}{{\rm{A}}}\times {\rm{100}}$$

where A is the concentration of analyte standards prepared in mobile phase, B is the concentration of blank (unspiked) sample, C is the concentration of analyte detected from the sample spiked before extraction.

In recovery experiments, the recovery value will not exceed 100% in theory, however the system error, operation error and other errors are inevitable in practice. So, the measured value (C-B) may be greater than the actual amount added (A), resulting in recovery values higher than 100%, which is a common phenomenon in method validation^22–24^. Generally, the recovery value of range of 85–115% can be acceptable, which means that the analytical error of the method is ±15%, and that is in full compliance with the requirements in analysis.

In our study, unspiked water samples and water samples spiked with 3 dyes at three concentration levels (0.4, 2 and 10 μg·L^−1^) were analyzed by using proposed method (n = 3). The results are listed in Table 2. The relative recoveries of dyes at three concentration levels were in the range of 89.4–100.3%, with RSDs within 3.5%. MG, CV and MB were detected in river water and influent wastewater samples (Table 3). These results indicated that the established method could be successfully applied to analysis of target dyes at trace level in real samples.

**Table 2:** Recoveries of spiked water samples (n = 3).

Analytes	Spiked (μg∙L^−1^)	R% (RSD %)				
		Drinking water	Running water	River water	Influent wastewater	Efluent wastewater
MB	0.4	96.1(2.8)	91.5(2.4)	94.8(2.2)	91.5(3.1)	91.3(3.5)
	2.0	96.3(2.9)	89.4(3.4)	96.2(4.5)	94.9(2.3)	93.6(2.2)
	10.0	100.3(2.3)	99.8(1.4)	96.1(2.1)	94.6(3.2)	97.6(0.8)
MG	0.4	96.2(1.2)	93.2(0.4)	86.1(1.3)	89.5(1.9)	89.8(1.1)
	2.0	94.4(1.2)	93.4(4.2)	91.3(2.5)	92.3(0.9)	93.1(1.6)
	10.0	96.9(2.5)	99.5(1.9)	92.4(3.3)	98.2(2.4)	94.0(1.1)
CV	0.4	92.4(1.4)	95.4(1.1)	95.3(1.8)	94.4(1.1)	90.9(0.3)
	2.0	91.4(2.4)	96.3(1.8)	98.2(2.2)	90.3(0.4)	93.1(1.2)
	10.0	99.2(1.3)	97.5(0.7)	92.1(0.9)	93.5(1.3)	98.5(2.7)

**Table 3:** Determination of MB, MG, and CV in real water samples (μg·L^−1^).

Analytes	Drinking water	Running water	River water	Influent wastewater	Effluent wastewater
MB	—	<LOQ	0.38	<LOQ	—
MG	—	—	0.20	0.13	—
CV	—	—	0.82	1.16	—

—: Not detected.

### Comparison of MSPE-IL-DLLME method to previous methods

The extraction effects of MG, CV and MB were significantly enhanced by the proposed MSPE-IL-DLLME coupled with HPLC–UV method. Furthermore, the current protocol can be evaluated by comparing with previously reported work from the viewpoints of extraction solvent, LODs, enrichment factor (EF), and recoveries etc. The extraction solvent using in DLLME is [Hpy]NTf_2_, which is more environmental and provides new insight in extraction techniques. According to the Table 4, the established method provides relatively more sensitive, higher EF and better recoveries than previous reports^3,25–28^. These merits make our method as a reliable tool in monitoring MG, CV and MB pollution in water samples.

**Table 4:** Comparsion of MSPE-DLLME-HPLC/UV with other methods for the determination of industrial dyes.

Methods	Analytes	Analytes in common	Matrix	Extraction Solvent	Volume (μL)	LOD (μg∙L^−1^)	Precision (RSD%)	EF	Recovery (%)	References
MSPE-HPLC/FLD	Malachite green, Gentian violet, Leuomalachite green, Leucogentian violet	Malachite green	Aquafarm water	—	—	0.09–0.22	2.7–6.5	91–95	87.0–92.8	3
MCPE-UV/Vis	Malachite green, Crystal violet, Rhodamine B	Malachite green, Crystal violet	Tap water; Wastewater	—	—	2.2–5.1	4.5–8.39	29.26–85.47	59.5–115.0	25
S-FF-DSPE-UV/Vis	Methylene blue	Methylene blue	Tap water; River water	—	—	2.5	2.9	135	99.0–109.0	26
DLLME-HPLC/UV	Malachite green, Crystal violet	Malachite green, Crystal violet	Upriver water	[C_8_MIM]PF_6_	80	0.030–0.086	7.6–9.4	254–276	71.7–97.2	27
SM-DLLME-UV/Vis	Malachite green	Malachite green	Textile industry wastewater	Decanoic acid -tetrahydrofuran	45500–500	4	1.8	52	85.3–108.6	28
MSPE-DLLME-HPLC/UV	Malachite green, Crystal violet, Methylene blue	—	Drinking water; Running water; Influent wastewater; Effluent wastewater; River water	[Hpy]NTf_2_	70	0.03–0.05	1.2–2.6	484–502	86.1–100.3	Represented method

## Conclusions

A novel sorbent based [Hpy]NTf_2_ functionalized MNPs was synthesized for the pretreatment through MSPE combined DLLME for the enrichment of 3 dyes in water. The obtained Fe_3_O_4_@SiO_2_@[Hpy]NTf_2_ adsorbents were characterized via FTIR, TGA, XRD and SEM. Under the optimized conditions, the method provided suitable analytical characteristics including extraction efficiency up to 86%, enrichment factor over 480, and LOD between 0.03–0.05 μg·L^−1^. Based on the results, we can concluded that Fe_3_O_4_@SiO_2_@[Hpy]NTf_2_ MNPs could be introduced as a novel sorbent for rapid extraction of dyes from aqueous solution.

## References

[1] Fu J (2016). Selective adsorption and separation of organic dyes from aqueous solution on polydopamine microspheres. J. Colloid Interface Sci..

[2] Chen J (2008). A core-shell nanoparticle approach to photoreversible fluorescence modulation of a hydrophobic dye in aqueous media. Chemistry..

[3] Chen, J. *et al*. Enhanced Performance of Magnetic Graphene Oxide-Immobilized Laccase and Its Application for the Decolorization of Dyes. *Molecules*. **22**, 10.3390/molecules22020221 (2017). 10.3390/molecules22020221 PMC6155931 28157159

[4] Oplatowska M (2011). The potential for human exposure, direct and indirect, to the suspected carcinogenic triphenylmethane dye Brilliant Green from green paper towels. Food Chem. Toxicol..

[5] Pierrard MA (2012). Malachite green toxicity assessed on Asian catfish primary cultures of peripheral blood mononuclear cells by a proteomic analysis. Aquat. Toxicol..

[6] Sun H, Wang L, Qin X, Ge X (2011). Simultaneous determination of malachite green, enrofloxacin and ciprofloxacin in fish farming water and fish feed by liquid chromatography with solid-phase extraction. Environ. Monit. Assess..

[7] Xu YJ (2012). Simultaneous determination of malachite green, crystal violet, methylene blue and the metabolite residues in aquatic products by ultra-performance liquid chromatography with electrospray ionization tandem mass spectrometry. J. Chromatogr. Sci..

[8] Lian Z, Wang J (2012). Molecularly imprinted polymer for selective extraction of malachite green from seawater and seafood coupled with high-performance liquid chromatographic determination. Mar. Pollut. Bull..

[9] Huang (2015). Determination of malachite green in fish based on magnetic molecularly imprinted polymer extraction followed by electrochemiluminescence. Talanta.

[10] Zhao J, Wei D, Yang Y (2016). Magnetic solid-phase extraction for determination of the total malachite green, gentian violet and leucomalachite green, leucogentian violet in aquaculture water by high-performance liquid chromatography with fluorescence detection. J. Sep. Sci..

[11] Afkhami A, Moosavi R, Madrakian T (2010). Preconcentration and spectrophotometric determination of low concentrations of malachite green and leuco-malachite green in water samples by high performance solid phase extraction using maghemite nanoparticles. Talanta..

[12] Jafarvand S, Shemirani F (2011). Supramolecular-based dispersive liquid-liquid microextraction: a novel sample preparation technique utilizes coacervates and reverse micelles. J. Sep. Sci..

[13] Huang P (2016). Trace determination of antibacterial pharmaceuticals in fishes by microwave-assisted extraction and solid-phase purification combined with dispersive liquid-liquid microextraction followed by ultra-high performance liquid chromatography-tandem mass spectrometry. J. Chromatogr. B.

[14] Cai MQ (2016). Planar graphene oxide-based magnetic ionic liquid nanomaterial for extraction of chlorophenols from environmental water samples coupled with liquid chromatography-tandem mass spectrometry. J. Chromatogr. A.

[15] Liu X (2014). Fe_3_O_4_@ionic liquid@methyl orange nanoparticles as a novel nano-adsorbent for magnetic solid-phase extraction of polycyclic aromatic hydrocarbons in environmental water samples. Talanta..

[16] Arain SA (2016). A new dispersive liquid-liquid microextraction using ionic liquid based microemulsion coupled with cloud point extraction for determination of copper in serum and water samples. Ecotoxicol. Environ. Saf..

[17] Vázquez MM, Vázquez PP, Galera MM, Moreno AU (2014). Comparison of two ionic liquid dispersive liquid-liquid microextraction approaches for the determination of benzoylurea insecticides in wastewater using liquid chromatography-quadrupole-linear ion trap-mass spectrometry: evaluation of green parameters. J. Chromatogr. A.

[18] Sun JN, Chen J, Shi YP (2014). Multiple functional ionic liquids based dispersive liquid-liquid microextraction combined with high performance chromatography for the determination of phenolic compounds in water samples. Talanta..

[19] Zhao P (2016). Solid-phase extraction combined with dispersive liquid-liquid microextraction and chiral liquid chromatography-tandem mass spectrometry for the simultaneous enantioselective determination of representative proton-pump inhibitors in water samples. Anal. Bioanal. Chem..

[20] Liang N (2016). Solid-phase extraction in combination with dispersive liquid-liquid microextraction and ultra-high performance liquid chromatography-tandem mass spectrometry analysis: the ultra-trace determination of 10 antibiotics in water samples. Anal. Bioanal. Chem..

[21] Li M, Yang L, Fang S, Dong S (2011). Novel polymeric ionic liquid membranes as solid polymer electrolytes with high ionic conductivity at moderate temperature. J. Membrane Sci..

[22] Yokley RA, Mayer LC, Huang SB, Vargo JD (2002). Analytical method for the determination of metolachlor, acetochlor, alachlor, dimethenamid, and their corresponding ethanesulfonic and oxanillic acid degradates in water using SPE and LC/ESI-MS/MS. Anal. Chem..

[23] Evans SE, Davies P, Lubben A, Kasprzyk-Hordern B (2015). Determination of chiral pharmaceuticals and illicit drugs in wastewater and sludge using microwave assisted extraction, solid-phase extraction and chiral liquid chromatography coupled with tandem mass spectrometry. Anal. Chim. Acta..

[24] Caballo C, Sicilia MD, Rubio S (2015). Enantioselective determination of representative profens in wastewater by a single-step sample treatment and chiral liquid chromatography-tandem mass spectrometry. Talanta..

[25] Elham G, Massoud K (2016). Application of Micro-cloud point extraction for spectrophotometric determination of Malachite green, Crystal violet and Rhodamine B in aqueous samples. Spectrochimi. Acta A.

[26] Negin FR, Farzaneh S (2015). Surfacted ferrofluid based dispersive solid phase extraction; a novel approach to preconcentration of cationic dye in shrimp and water samples. Food Chem..

[27] Zhang Z (2016). Determination of malachite green and crystal violet in environmental water using temperature-controlled ionic liquid dispersive liquid-liquid microextraction coupled with high performance liquid chromatography. Ana. Methods.

[28] Sanaz J, Farzaneh S (2011). Supramolecular-based dispersive liquid–liquid microextraction: A novel sample preparation technique utilizes coacervates and reverse micelles. J. Sep. Sci..

